# On the Glucogenetic Functions of the Liver

**Published:** 1855-11

**Authors:** 


*On the Glucogenetic Functions of the Liver.*—It has been generally acknowledged, for some years past, that Bernard's discovery of the formation

---

of sugar in the liver, has been demonstrated by incontestible experiments. The *Medico-Chirurgical* Reviewers, and the sceptical Todd, and Bowman, so slow to accept truths that have other than an English origin, long ago accepted Bernard's results, which have since been substantiated by experimenters in every country. "There can be no doubt," say Todd and Bowman, "that sugar is formed in the liver independently of the food," (*Phys. Anat.* p. 605). Opinions varied as to how sugar was formed, and as to what became of it after its production, but physiologists accepted the facts, however they differed in their interpretation.

It was a bold step, therefore, in M. Figuier, a young and promising physiologist and chemist, of Paris, author of an *History of Modern Discoveries*, to deny altogether the conclusions of Bernard, and facts which are now taught in all schools of medicine. M. Figuier presented his lengthy and well-written memoir to the French institute, on the 29th of January last. He attempted to prove that sugar was a normal ingredient of the blood, and was simply stored up in the liver. M. Bernard replied by a lecture at the College of France, in which he demonstrated, by a careful repetition of his experiments, that in animals fed on meat, there was no sugar in the blood of the portal vein, whereas the blood of the hepatic veins abounded in it. Consequently, sugar was formed somewhere between these points, that is, in the liver. The journals having published some experiments in favor of the views of the professor of the school of pharmacy, M. Figuier, on the 12th of March, M. Bernard presented to the Institute a paper by the eminent physiologist of Leipsig, Professor Lehmann, entirely corroborating his own experiments. On March 26th, M. Figuier rejoined by a detail of experiments, proving that the blood of the portal vein does contain sugar even in animals fed on meat. We have laid before our readers, in a recent number, (*ante*, p. 126) an analysis of the experiments of Lehmann and Figuier, extracted from our cotemporary the *Medical Times and Gazette.* The latter part of that article contains an abstract of the next document in the polemic, the history of which, we now briefly sketch: M. Bernard, growing indignant, contemptuously denies the accuracy of the statements of M. Figuier, and asks to be discharged from the committee to which his papers were referred. On the 16th of April, M. Leconte, who has latterly conducted M. Magendie's experiments, laid before the Academy the details of the analyses of the blood of five dogs, which he had sacrificed in M. Bernard's behalf. At the same meeting, M. Poggiale, professor of chemistry at the military school of medicine, at Val-de-Grace, communicated two series of experiments: the first, on bitches, proved that the lacteal secretions contained sugar, when an animal was fed on meat diet; the second, on dogs, confirmed the conclusions of Bernard in respect to the absence of sugar in the portal veins, and its presence in the hepatic veins. M. Poggiale also demonstrated that meat contained no sugar, and attempted to account for the origin of this principle in the liver, by a supposed transformation of fatty matters.

All these documents were referred to a committee, MM. Dumas, Pelouze and Rayer. On the 13th of June, Dumas brought in a report. As an authoritative decision on this important point, we translate this report *verbatim* from the August (1855) number of the *Archives générales de Médicine:*

"The Academy has desired MM. Pelouze, Rayer, and myself, to report on the recent experiments of MM. Figuier, Poggiale, and Leconte, in regard to the true functions of the liver. Your committee has thought best to restrict their duties to a simple verification of facts, setting aside all theoretical considerations whatever. It has endeavored to perform this task with the utmost precision attainable in the present state of science.

"One of our colleagues, M. Bernard, has announced, in conjunction with M. Barresevil, that the liver contains a notable quantity of sugar. M. Bernard has proved that sugar exists in the livers of all animals; that its presence is consequently an indication of the nature of the function of this important organ.

"Thus far, the noble observations of M. Bernard and the consequences he deduces from them are contested by no one; they constitute one of the greatest advances of modern physiology. But whence comes this sugar that is constantly found in the liver? What is its use? Here opinions diverge, difficulties present themselves, and experiments are contradictory.

"M. Bernard thinks that sugar is formed in the liver. He does not deny that it is also formed during digestion, in the stomach, at the expense of the amylaceous constituents of the food, not that glucose or its analogue enters the veins of the stomach and intestines; but he claims that, in addition to this occasional sourse, of glucose in the blood, there is a permanent and special source, at which sugar is elaborated; that sugar, in short, is fabricated in the liver.

"This formation is demonstrated, he asserts, by the absence of sugar in the vena portæ of an animal restricted to a meat diet, and its presence in the hepatic veins of the same animal.

"M. Figuier raises numerous objections to this doctrine. He first advances an opinion already offered by Mialhe, that it is more natural to consider the liver an eliminative organ, like the kidneys, than a creative organ. According to his hypothesis, the liver is a regulator of the composition of the blood, and arrests in its passage the sugar derived from digestion, when it is in excess, just as it arrests certain metallic poisons, and restores the sugar to the blood, little by little, when, during the hours of fasting, the proportion of sugar falls below the mean.

"The function attributed to the liver by M. Bernard, is based on four principal facts: 1. The constant presence of sugar in the liver of carnivorous and herbivorous animals; 2. The not less constant presence of sugar in the veins leading from the liver; 3. The absence of sugar in the blood of the portal veins of animals fed on meat; 4. The temporary presence of sugar in the portal vein during the digestion of saccharine or feculent food. It was the duty of your committee to examine whether these data were contested, and, if they were, on what grounds.

"The first and fourth facts were not questioned. It is admitted that the liver always contains sugar, even in carnivorous animals; it is equally admitted that the portal veins contain sugar during the digestion of amylaceous matters. It remains to inquire whether the portal vein contains sugar in animals fed exclusively on meat.

"On this point the experiments of your committee appear perfectly decisive; *it could find no appreciable traces of sugar in the blood of the portal vein of a dog fed on raw meat.* It still remains to determine whether the hepatic veins contain sugar independently of alimentation by vegetable food; if, lastly, the hepatic veins contain sugar when there is none in the blood of the portal veins.

"It suffices, to clear up these points, to examine the blood of the portal and hepatic veins on the same animal, as M. Bernard has done, the animal being engaged in digestion, after a meal of raw meat, eaten either after protracted abstinence, or after several days of animal regimen.

"Your committee has satisfied itself, that, in an experiment conducted on these conditions, the portal vein contained no sugar, whereas the presence of sugar was very manifest in the hepatic veins.

"As the difficulty centered on this point: Whether the blood of the portal vein of an animal fed on meat exclusively, contained sugar during digestion? your committee has examined, with all possible care, the products extracted by M. Figuier from the vena portæ of an animal sacrificed under these circumstances, in which M. Figuier believed that he had detected sugar by the Fromberz test (copper and potash.) Your committee could find no sugar in these products, employing, it is true, the fermentation test.

"Thus all the facts announced by our colleague, M. Bernard, in relation to the function he attributes to the liver, have been verified by us, and we cannot refrain from expressing our admiration for the rare skill with which the learned physiologist has demonstrated them.

"Your committee does not feel called on to discuss this question in its doctrinal aspect. Does the liver produce sugar? Does it fabricate sugar at the expense of the albuminous constituents of the blood? Is sugar a product of digestion or an elaboration of the elements of the blood during its circulation which is masked by some foreign principle until it reaches the liver and is liberated? These questions assuredly merit discussion; but experiment alone will solve them; and it is with pleasure that we observe the perseverance of the young *savants* who have undertaken them.

"Thus far, the doctrine by our colleague is not invalidated. We desire to say to those engaged in researches on this subject, that they *should not accord absolute confidence to such chemical reäctions as, for example, are obtained by solutions of tartrate of copper and potash. All these phenomena of reduction and coloration are uncertain and deceptive.* When we cannot isolate sugar in nature, we should at least assure ourselves of its presence by fermentation and the development of carbonic acid produced thereby, as was done by the academic committee which originally examined the investigations of M. Bernard.

"Your committee, then, establishes:

"1. That sugar was not present in appreciable quantities in the blood of the portal vein of a dog fed on meat.

"2. That sugar was easily detected in the blood of the hepatic veins of the same dog, the blood being collected from the different veins at the same moment.

"As MM. Figuier, Poggiale, and Leconte have published their papers, the Academy, in accordance with its rules, will not pronounce on the respective merits of these memoirs. Your committee, however, have felt it their duty to lay before you the result of its experiments on the question which is the essential point of the subject these learned gentlemen have examined."—*Virginia Med. and Surg. Journal.*

